# Relationship of Mothers’ Psychological Status with Development of Kindergarten Children

**Published:** 2016

**Authors:** Firoozeh SAJEDI, Mahbobeh AHMADI DOULABI, Roshanak VAMEGHI, Mohammad Ali MAZAHERI, Alireza AKBARZADEHBAGHBAN

**Affiliations:** 1 Pediatric Neurorehabilitation Research Center, University of Social Welfare and Rehabilitation Sciences, Tehran, Iran; 2 PhD Candidate of Pediatric Neurorehabilitation research Center, University of Social Welfare and Rehabilitation Sciences,Tehran, Iran; 3 Psychology Department, ShahidBeheshti University, Tehran, Iran; 4 Ph.D in Biostatistics, Proteomics Research Center,Department of Basic Sciences, School of Rehabilitation, ShahidBeheshti University of Medical Sciences, Tehran, Iran

**Keywords:** Depression, Anxiety, Stress, Developmental delay

## Abstract

**Objective:**

Given the importance of children’s development and the role of psychological status of mothers in this regard, this study investigated the relationship of mothers’ psychological status (stress, anxiety and depression) with the development of children aged 36-60 months.

**Materials & Methods:**

This descriptive study was performed on 1036 mothers and their children, aged 36 to 60 months, in different kindergartens in Tehran City, Iran, in 2014-2015. Participants were selected through multi-stage random sampling. The following instruments were used in this study: A demographic and obstetric specification questionnaire, children specification questionnaire, the Beck Depression Inventory, Spiel Berger Test, Perceived stress questionnaire and the Ages and Stages Questionnaire to determine the status of the children’s development. Data were analyzed using SPSS20 software, Mann-Whitney; independent t-test and logistic-Regression model were used.

**Results:**

The prevalence of developmental delay in children aged 36-60 months was 16.2%. The independent t-test showed a relationship between maternal stress and developmental delay in children. The Mann-Whitney test revealed a significant relation between mothers’ depression level and developmental delay in their children. There was a significant relation between trait anxiety and developmental delay in children. Moreover, a significant relation was found between maternal stress and developmental delay in fine motor skills. The logistic regression model showed a significant relationship of child gender, economic and social states with developmental delay.

**Conclusion:**

This study showed mothers’ psychological status probably is an effective factor in developmental delay. The assessment of mothers’ psychological status is suggested for early interventions.

## Introduction

The years from birth to five years of age is a critical time in the development of cognitive, emotional, physical, social, language, and behavioral skills. Early child development includes language, social, kinetic, cognitive and emotional areas.This period has determinant impact on early learning, risk of physical and mental diseases, educational achievement, economic participation, social role of citizenship, and health (1-3). According to estimations, 200 million children are not completely developed worldwide, and this issue has huge impacts on their health and society at wider scale (4). Developmental delay is defined as a significant functional delay in two or more developmental domains (5). The developmental delay is a priority in medical and health systems, even in advanced countries like US (6). Worldwide, 15%-18% of children have speech, learning, and emotional-behavioral disorders, and 25% are with serious mental-social problems (7-8). The prevalence of developmental disabilities has been reported as 15 %in US, 15% in Jamaica, 8% in Bangladesh, 15% in Pakistan, 1.5-2.5% in India (among children<2 years), up to 10% in Iraq, 3.3% in Brazil, and 12.5% in Holland (4,6,9-12). Eight percent of preschool-aged children (birth to six years) have developmental disorder in one or more areas, indicating the importance of time in diagnosis and treatment of developmental disorders (13). Domestic studies in Iran have reported 7%-26.3% prevalence of developmental delay in Iranian cities (14, 15-18), with 18% incidence among children aged 4-60 months in Tehran (19). A wide spectrum of causes, including social factors, are associated with the incidence of developmental delay; therefore, identification of these risk factors is very essential (,^10^,^11^, ^20^). The significant role of families in the development and growth of children has been recognized since 1930 (21). Accordingly, a safe family setting allows the child to get benefit from his or her maximum developmental potential. Evidence suggests that the keys to optimal development of children are safe and secure connection with caregivers, and support and affection from them (22,23). Different studies have indicated the effects of demographical variables of life style (such as mother’s mental health, stressful events in mother’s life, social and economic states, stressful jobs, cultural matters, drug abuse by parents, child abuse, low social supportfrom mother, and inappropriate parental behaviors) on children development (11, 21, 24-27). Mother’s mental problems affect the quality and quantity of childcare; in addition, mother’s negligence towards learning stimulants in their children leads to sever irritability, and learning and behavioral problems in them (28,29). Koutra et al., reported a correlation of mothers’ depression with delayed development of children in the gross motor and cognitive domains of development (30). Mother’s depression limits maternal responsibilities in meeting the social and emotional needs of the children and results in increased behavioral problems in them (31). Additionally, the infants of mothers with depression have higher degrees of stress response in form of increased heart rate and cortisol level (32). There is a strong relationship between mothers’ experience of stressful events and developmental delay in their children, directly related to behavioral, mental, and emotional problems in them (33). The children of mothers with depression were more prone to depression, educational failure, and poor language, communicational, and emotional skills (31). Thus, identification of high-risk mothers and children, and performing preventive interventions are the best and most reasonable measures (12, 24). Given the importance of development in children, the effect of mothers’ mental state on this phenomenon, and significance of timely and appropriate interventions, this study investigated the relationship of mothers’ psychological factors (stress, anxiety, depression) with developmental delay in children aged 36-60 months.

## Material & Methods

This cross-sectional descriptive study was conducted from April 21, 2014 to February 20, 2015 in kindergartens across Tehran, Iran. The sample size included 1,036 mother-child pairs (201 children aged 36 months, 201 aged 42 months, 214 aged 48 months, 200 aged 54 months, and 220 aged 60 months), among which the eligible ones were included in the study. The eligible subjects were children living with their both parents who have not experienced any serious stressful and unpleasant events (such as loss) since six monthsbefore the study, and children with no diagnosed developmental disorder.

**Data Collection**

In Iran, kindergartens are under the supervision of the Welfare Organization with three centers in Tehran (north, central, south). Each of these welfare centers covers kindergartens of certain regions across the city. In the first stage, stratified sampling technique was used and then some regions were selected from each category using simple random sampling method, taking the number of regions affiliated to each welfare center into account. In this way, two, seven, and three regions were selected from the welfare north, central, and south centers, respectively. Among the selected regions, some kindergartens were chosen using simple random sampling method and proportional to the total number of kindergartens. Sampling in the selected kindergartens was purposive, considering the research criteria. The data collection instruments included parents-child demographic inventory, socioeconomic questionnaire, and Beck Depression Inventory for measuring the mothers’ depression levels Perceived Stress Scale (PSS-14), Spiel Berger’s State Anxiety Scale, and Ages and Stages Questionnaire (ASQ) were used for measuring maternal depression, maternal state-trait anxieties, and children’s developmental progress, respectively. Moreover, the content validity method was employed to validate demographic and socioeconomic inventories, scientific resources and opinions of experts are used. The demographic inventory included parents’ general information (age, educational attainment, job, gravidity and parity, and history of abortion). The socioeconomic status was assessed by studying income, Price square feet residential ground, hosing number of family, number of cars, and personal computer. By using content validity and test-retest (The correlation coefficient was 93%- 97% from 10 checklists), the validity and reliability of the demographic, socioeconomic status, and child specification questionnaire were determined. For screening of depression the Beck Depression InventoryII (BDI-II) was used in addition to 21 items scoring from 0 to 63 most commonly utilized for measurement of depression.

**Beck Depression Inventory-II (BDI-II)**

In order to determine depression levels, the scores 0–9, 10–18, 19–30, 31–40, and 41–63 respectively indicated normal, mild, moderate, severe, and extremely severe depression. Different studies have proved reliability of the test (34-36). In Iranian population, the internal consistency was confirmed with Cronbach’s alpha of 0.87 and reliability coefficient was found at 0.74 (37). The reliability of the questionnaire was measured as 0.85, using Cronbach’s alpha.

**Perceived stress questionnaire :( PSS-14)**

To assess general perceived stress in the recent month, perceived stress questionnaire was prepared by Cohen et al. in 1983 (38). By this questionnaire, thoughts and feelings about stressful events control of overcoming and dealing with psychological pressure and experienced stresses were assessed. In addition, the risk factors in behavioral disorders and indicated stressful relationship processes were examined using this scale. In various countries, this questionnaire has frequently been used, normalized and translated into different languages. The 14-item version of the questionnaire was used in this study. By using the 5 point Likert scale beginning with “Never=0” and ending with “very often=4” scoring was done. Seven positive items indicated well-adopted individuals with stressful factors, and seven negative items indicated inability to cope with stress. The highest and the lowest score was 56 and 0, respectively. The greater perceived stress was shown by higher scores. In 3 studies Cronbach’s alpha for this scale was in the range of 0.84 to 0.86 (38). Bastani et al. reported Cronbach’s alpha as 0.81 for the scale (39). In our study, Cronbach’s alpha was 0.90.

**Spielberger’s inventory**

This study used Spielberger’s inventory to measure maternal anxiety. This scale consists of two groups of items. The State Anxiety and Trait Anxiety tests are comprised of 20 questions each, which measure anxiety symptoms. Response to each item is scored in arrange of 1-4. In this test, the minimum and maximum anxiety scores are 20 and 80, in which 20-40, 41-60, and 61- 80 indicate mild, median, and sever anxieties. TheSpielberger’s scale is a valid inventory for measuring anxiety. Its reliability has also been determined in several studies. In Iran, reliability of these two scales has been computed as 0.91 and 0.95 in Tehran and Mashhad, respectively (40-46). In the present study, the reliability of trait and state anxieties was obtained as 0.85 and 0.90, using Cronbach’s alpha.

**ASQ Questionnaire**

Currently the most widely used test is ASQ. Its specificity is 95% and 90% and sensitivity 75% and 100% for high risk group and the community group, respectively (47). Nineteen different questionnaires that can screen developmental status of children from four to 60 months in five different domains: communication, gross motor, fine motor, problem solving and personal-social skills were used and the validity of this test varies from 76% to 88%. Based on what the child can or cannot do, each domain is evaluated by six questions. In order to be representatives of a developmental quotient of 75–100%, they are selected. To indicate that the child does the special behavior of this item, the answer of parents to each question is “yes” and to indicate an occasional or emerging response, ‘sometimes’’ and ‘‘not yet’’ to indicate that their child does not yet do the behavior, with a respective score of 10, 5 or 0 points. Final score in each domain is compared to cut-off points of these guidelines, so scores of each item summed. The abnormal and referral for further evaluation is the score on any domain below the cut-off point or more than two standard deviations below the mean of the reference group (48-52). ASQ is a reliable tool with Cronbach’s alpha of 0.86 and reliability of 0.93 for Iranian children A reliable tool with Cronbach’s alpha of 0.86 and reliability of 0.93 for Iranian children is ASQ (53). The reliability of this scale in present study was obtained as 0.88, using the test-retest method. After securing the required permissions and explaining the research objectives to the authorities and in structures to enlist their cooperation, the researcher attended the research setting and obtained parents’ written informed consents for participation in the study. Maternal and child demographic inventories, perceived stress inventory, Spielberger’s scale, Beck’s checklist, and ASQ (proportional to child’s age) were given to themothers to complete at home in four days. After returning the questionnaires by the mothers, the researcher calculated ASQ scores by taking the determined cut-off (proportional to child’s age) into account, and declared the results to the parents. In case of obtaining a score lower than cut-off, the case was referred to a specialized assessment center.

**Data analysis**

In this stage, due to the ordinal nature of response variables (anxiety and depression) and isolation of groups from each other, the Mann-Whitney test was used. In addition, because of the normality of response variable (stress) and isolation of groups from each other, independent t-test and logistic regression model were employed. The significance level of tests was considered as P=0.05.

## Results

**Fig 1:**
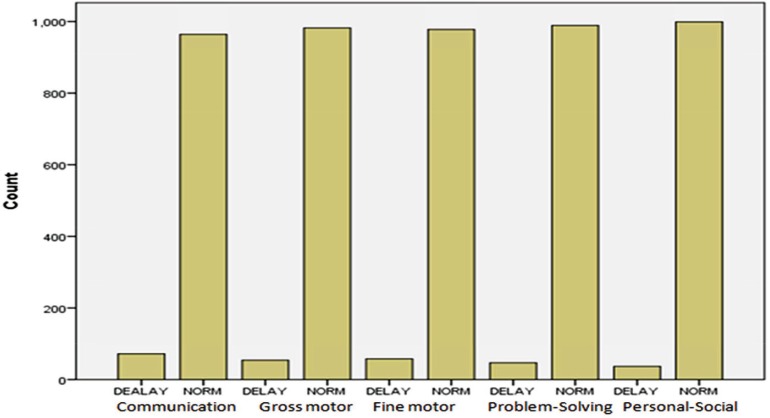
Frequency of developmental delay in children aged 36-60 months

The mean ages of mothers in children with normal development (first group) and developmental delay (second group) were 31.69 (5.38) and 31.21 (5.65) yr, respectively. On average, parents of the first and second groups had 11.77)4.63) and 11.92 (4.20) yr of formal education, respectively. The gravidity in these groups was 1.90 (1.5) and 1.78 (0.86), and the parity was 1.70 (0.97) as well as 1.58 (0.72), respectively. There was no statistically significant difference between the two groups in these regards. Among the subjects, 51.9% and 48.1% were girl and boy, respectively. The degree of developmental delay in children was 16.2%. Figure 1 shows the degree of developmental delay in each developmental area (Figure1). The maternal stress in mothers of the first and second groups was 28.28 (6.60) and 30.01 (6.87), respectively. The independent t-test showed a significant relationship between maternal stress and developmental delay [in children] (P=0.002). Moreover, a significant relation was found between maternal stress and developmental delay in fine motor skills (P=0.004) (Table1). 17.6% of mothers suffered from moderate to extremely severe depression. The relationship of developmental delay prevalence in five developmental areas with maternal depression is presented in Table 2. Accordingto the Mann-Whitney test, there was a significant correlation between mothers’ depression levels and children’s developmental delay. Table 2 also shows a significant relationship between maternal depression and developmental delay in communication and gross motor skills (P=0.0001), as well as with fine motor, and personal and social problem solving skills (P=0.001). The majority of samples (84.3%) had mild state anxiety. According to Table 3, 95.2% of the subjects had mild to severe trait anxiety, and no significant relationship was found between state anxiety in mothers and developmental delay in children; whereas, there was a significant correlation between trait anxiety in mothers and developmental delay in children (P=0.011). The logistic regression showed a correlation between the child gender and developmental delay, especially among girls with 94% higher probability (P=0.0001 and OR=1.94). Moreover, socioeconomic status was also related to developmental delay in children (P=0.001 and OR=0.396). However, the number of pregnancies, parity, and abortion, the age of mother, and father, the education of mother, and father had no correlation with developmental delay.

## Discussion

In this study, a significant correlation was observed between maternal depression, stress, and trait anxiety with the risk of developmental delay in children aged 36-60 months. The mental state of parents directly affects the development of children (54), in consistent with our study. Additionally, increasing number of evidence suggest that poor mental state of mothers negatively affect the children’s mental development (30). According to the studies, mental disorders have 20% prevalence in women, and depression is one of the most significant psychological problems and the most common type of mood disorder in about 25% of women in lifetime. The prevalence of depression among women in different cultures is relatively two times higher than men. In addition, 12-month prevalence rate of anxiety disorders has been reported as 17% (55,56). Maternal depression may lead to cognitive and language problems, poor social skills, and behavioral problems in early childhood. Parental depressioncan affect the child’s capability in social interactions and object recognition, which can be diagnosed from the second month after birth. In addition, relative to children of non-depressed mothers, the children of mother with depression undergo higher levels of stress, associated with increased heart rate and cortisol level in blood (30). In Pakistan, the children of depressed mothers are six times more prone to emotional development disorders, and there is correlation between maternal depressions with developmental delay in the gross, fine, language and cognitive development (57). Children of depressed mothers scored lower in fine and gross motor and personal-social skills, and in their general development (58). Moreover, Koutra et al., reported a correlation of mothers’ depression with delayed development of children in the gross motor and cognitive domains of development (30), in consistent with our results. The severity and duration of maternal depression increase children’s behavioral problems and retards their vocabulary development (59). A strong relationship between mothers’ experience of stressful events and children’s developmental delay, in that poor mental state and performance of parents are explicitly correlated with behavioral, mental, and emotional problems in children (33). Sever maternal anxiety results in irreparable damages to mother-child relationship, decreases motherhood capabilities, and probably leads to abnormal development due to negative reactions (60). In addition, the probability of having children with Attention Deficit Hyperactivity Disorder (ADHD), irritability, low weight, and eating and sleeping problems is greater in mothers with high anxiety (61). The children of mothers with anxious mood develop greater cognitive, social, emotional, and behavioral problems relative to the children of normal mothers (62). These results can emphasize the quality and extent of mother-child interactions (57, 63). Some evidence in developing countries imply that major depressions have negative impact on motherchild interactions, which is due to the negative effects of mother-child sense of security and attachment (64, 65). For example, the symptoms of depression and itsassociated negative self-perceptions will lead to lower child support, lower optimism, being influenced by inconsistent expressions, lower irritability, and lack of maternal responsiveness (66). In addition, the child will be kept from expanding his/her knowledge and building up a mental relationship with his/her mother (67). In this regard, children of anxious mothers are not supported by their mothers in activities and adjustment in different situations, and thus have problem in communicating and playing with peers. Moreover, children of anxious mothers are more prone to depression, anxiety, and mood disorders (68, 69). As said earlier, mother’s mental problems can affect the quality and quantity of childcare. In addition, the lack of attention to the child and learning stimulants leads to learning problems; sever irritability, and behavioral problems in the child (29, 70). It is assumed that sadness, irritability, and social perceptions, as characteristics of depressed women, risk their capabilities in creating a responsive and sensitive environment for their children (71-73). Children with more opportunity in discovering the surrounding world and gaining positive experiences during their early childhood development (ECD) are more capable of extending their cognitive abilities, learning from life experiences, and using them in similar situations (74). On the other hand, parenting styles have fundamental effects on child’s development. Parent-child interactions during infancy and early childhood are a cornerstone of child’s trust, which as an essential factor, encourages the child to discover his/her surrounding world and employ these discoveries in complete safe environment (75). Parental behaviors such as positive empowerment, and warm, emotional, and kind behaviors, as well as the use of disciplinary strategies consistent with the less behavior-specific problems in child will result in positive scientific capabilities and relationships with peers (76). Children of more sensitive and responsive mothers have better electrical activity in the left frontal lobe, indicating positive strategies and feelings; whereas, the electrical activity of children of less responsivemothers is concentrated on the right frontal EEG, indicating negative emotions, fear, and mood (77). However, Keim et al. showed that such characteristics as maternal anxiety, and prenatal and postpartum stress are not associated with significant negative impact on the child’s cognitive development (78). In addition, McCarthy Scales of Children Abilities did not show any correlation between maternal depression and cognitive development in children aged five yr. Moreover, no correlation was observed between the two mother groups with depression time (before, during, or after pregnancy), duration of depression, child’s gender, and socioeconomic factors (79). In contrast to some studies, Campbell et al. reported that despite postpartum depression, mothers play their motherhood role well enough and depression is not inevitable in them (80). The inconsistencies in the results of studies may be caused by difference between times of sampling studied; this is a very effective factor, because in some articles these studies were performed at postpartum. The other probable causes were in sample sizes, different sampling methods and developmental tools. It is reported that 36-month-old children start becoming more sensitive to their parents’ emotions with time, and their kinetic development is directly affected by the mothers’ mental health. Toddlers show their feelings by actions to compensate for their verbal incapability in doing so. Therefore, children of mothers with mental health problems are more prone to behavioral and emotional disorders (54). With respect to the relationship of mother’s mental health with different areas of development, it seems that as compared with the depression alone, the accumulation of adverse conditions (such as low levels of SES, and sever and chronic depression of mother) has greater impact on the child’s cognitive development (81). Murr et al. represented long-term adverse impacts of maternal depression on cognitive and behavioral development, especially in boys (82). children of depressed and low-income mothers are five times more prone to delay in language development. This is because the depressed mothers are more concern about financial restrictions and thus have less vocalizing interaction with their children. إoys weremore susceptible to delayed language development (57), in consistent with the present results. As limitation of this study, our findings are limited to the mothers with kindergarten children.

**Table 1:** Relation of Mothers Stress and Developmental Delay with five Domains of Development

**Domains**	**Communication**		**Fine Motor**		**Gross Motor**		**Problem-Solving**		**Personal social**	
	**Delay** **(+)**	**Delay** **(-)**	**Delay** **(+)**	**Delay** **(-)**	**Delay** **(+)**	**Delay** **(-)**	**Delay** **(+)**	**Delay** **(-)**	**Delay** **(+)**	**Delay** **(-)**
**MeanStress**	**28.611** **(6.7)**	**28.57** **(6.6)**	**31.01** **( 6.6)**	**28.42** **(6.7)**	**29.22** **(6.5 )**	**28.52** **(6.7)**	**28.36** **( 6.8)**	**28.57** **( 6.6)**	**28.08** **( 7.9)**	**28.57** **(6.6)**
**Result of In depended T test**	***P*** **=0.956**		***P*** **=0.004**		***P*** **=0.453**		***P*** **=0.288**		***P*** **=0.0651**	

**Table 2:** Relation of Mother’s Depression and Developmental Delay with Five Domains of Development

**Domains**	**Communication**		**Fine Motor**		**Gross Motor**		**Problem-Solving**		**Personal social**	
**Depression**	**Delay** **(+)**	**Delay** **(-)**	**Delay** **(+)**	**Delay** **(-)**	**Delay** **(+)**	**Delay** **(-)**	**Delay** **(+)**	**Delay** **(-)**	**Delay** **(+)**	**Delay** **(-)**
**None**	**21** **(3.8)**	**530** **(96.2)**	**22** **(4)**	**964** **(96)**	**16** **(12.9)**	**535** **(97.1)**	**17** **(3.1)**	**534** **(96.9)**	**11** **(2)**	**540** **(98)**
**Mild**	**26** **(8.60**	**277** **(91.4)**	**15** **(5)**	**288** **(95)**	**19** **(6.3)**	**284** **(93.7)**	**11** **(3.6)**	**1** **(96.4)**	**13** **(4.3)**	**290** **(95.7)**
**Moderate**	**18** **(12.3)**	**128** **(87.7)**	**16** **(11)**	**130** **(89)**	**14** **(9.6)**	**132** **(90.4)**	**12** **(6)**	**134** **(91.8)**	**7** **(4.8)**	**139** **(95.2)**
**Sever**	**7** **(23.3)**	**23** **(76.6)**	**5** **(16.7)**	**25** **(83.3)**	**5** **(16.7)**	**25** **(83.3)**	**6** **(20)**	**24** **(80)**	**5** **(16.7)**	**25** **(83.3)**
**Extremely Sever**	**0** **(0)**	**6** **(100)**	**0** **(0)**	**6** **(100)**	**0** **(0)**	**6** **(100)**	**1** **(16.7)**	**5** **(83.3)**	**1** **(16.7)**	**5** **(83.3)**
**Total**	**72** **(6.9)**	**964** **(93.1)**	**58** **(5.6)**	**982** **(94.4)**	**54** **(5.2)**	**982** **(94.8)**	**47** **(4.5)**	**989** **(95.5)**	**37** **(3.6)**	**999** **(96.4)**
**Result of** **Man-Witney test**	***P*** **=0.0001**		***P*** **=0.001**		***P*** **=0.0001**		***P*** **=0.001**		***P*** **=0.001**	

**Table 3:** Relation of Mothers Anxiety and Developmental Delay with Five Domains of Development

**Domains**		**Communication**		**Fine Motor**		**Gross Motor**		**Problem-Solving**		**Personal social**	
**Anxiety**		**Delay** **(+)**	**Delay** **(-)**	**Delay** **(+)**	**Delay** **(-)**	**Delay** **(+)**	**Delay** **(-)**	**Delay** **(+)**	**Delay** **(-)**	**Delay** **(+)**	**Delay** **(-)**
**Trait-** **Anxiety**	**Mild**	**7** **(14)**	**43** **(86)**	**3** **(6)**	**47** **(94)**	**5** **(10)**	**45** **(90)**	**2** **(4)**	**48** **(96)**	**2** **(4)**	**48** **(96)**
	**Moderate**	**62** **(6.9)**	**837** **(93.1)**	**52** **(5.8)**	**847** **(94.2)**	**49** **(5.5)**	**850** **(94.5)**	**43** **(4.8)**	**856** **(95.2)**	**34** **(3.8)**	**865** **(96.2)**
	**Sever**	**3** **(3.4)**	**84** **(96.6)**	**3** **(3.4)**	**84** **(96.6)**	**0** **(0)**	**87** **(100)**	**2** **(2.3)**	**85** **(97.7)**	**1** **(1.1)**	**86** **(98.9)**
	**TOTAL**	**72** **(6.9)**	**964** **(93.1)**	**58** **(5.6)**	**978** **(94.4)**	**54** **(5.2)**	**982** **(94.8)**	**47** **(4.5)**	**989** **95.5)**	**37** **(3.6)**	**999** **(96.4)**
	**Result of** **Man-Witney** **Test**	***P*** **=0.028**		***P*** **=0.007**		***P*** **=0.435**		***P*** **=0.480**		***P*** **=0.278**	

**In conclusion**, poor psychological state of mother may have negative impacts on the child’s development; therefore, regarding the significance of optimal child development, adoption of appropriate strategies and interventions for primary prevention, diagnosis, and assessment of that problem seems essential. In addition, better understanding of the effect of mothers’ psychological state on child’s development may lead to significant outcome in an attempt to perform required preventive interventions.
